# Objective and subjective evaluation of a passive low-back exoskeleton during simulated logistics tasks

**DOI:** 10.1017/wtc.2023.19

**Published:** 2023-09-19

**Authors:** Lukas Mitterlehner, Yasmin Xinyue Li, Matthias Wolf

**Affiliations:** Institute of Innovation and Industrial Management, Graz University of Technology, Graz, Austria

**Keywords:** exoskeletons, industrial engineering, objective/subjective evaluation

## Abstract

Musculoskeletal disorders remain the most common work-related health problem in the European Union. The most common work-related musculoskeletal disorder reported by workers is backache, especially in the logistics sector. Thus, this article aims to evaluate the effects of a commercial passive low-back exoskeleton during simulated logistics tasks. Thirty participants were recruited for this study. Typical logistics tasks were simulated in a laboratory environment. Cross-over research design was utilized to assess the effects of the exoskeleton on heart rate, trunk inclination, trunk acceleration, throughput, and perceived exertion. Also, usability and acceptance were obtained using a custom questionnaire. We found mostly non-significant differences. Effects on throughput varied widely between workplaces. Usability ratings were poor and acceptance moderate. The study suggests that a holistic evaluation and implementation approach for industrial exoskeletons is necessary. Further, prior to exoskeleton implementation, workplace adaptation might be required.

## 1. Introduction

Work-related musculoskeletal disorders (WMSDs) are impairments of bodily structures, such as muscles, joints, tendons, ligaments, nerves, bones, and the localized blood circulation system that are caused or aggravated primarily by work and by the effects of the environment in which work is carried out (Podniece, 2008). The sixth wave of the European Working Conditions Survey revealed that roughly three out of five workers reported WMSD complaints (Eurofund, 2017). Thus, they remain the most common work-related health problem in the European Union (European Agency for Safety and Health at Work et al., 2020). The predominant WMSD is backache, affecting 43% of all workers (Podniece, 2008).

Literature suggests that WMSDs are caused by a combination of (1) physical factors (e.g., high physical demand, repetitive motion, and awkward or static posture), (2) psycho-organizational factors (e.g., monotonous tasks, high work rate, and time pressure), and (3) individual factors (e.g., medical history, age, and tissue tolerance) (Malchaire et al., 2001; da Costa and Vieira, 2010). On the sector level, backache is most prevalent in the agriculture, construction, manufacturing, and transportation and logistics sector (European Agency for Safety and Health at Work et al., 2020). In these sectors, workers frequently remain exposed to the aforementioned risk factors. While the implementation of technical and organizational ergonomic measures could potentially solve many problems, it is often not feasible due to workplace characteristics that require flexibility and human competences (de Looze et al., 2015; Maurice et al., 2020; Kermavnar et al., 2021).

In response, industrial exoskeletons emerged recently to reduce muscular stress and prevent WMSDs (de Looze et al., 2015; Howard et al., 2019; Peters and Wischniewski, 2019). The increasing interest in industrial exoskeletons is reflected in the rapidly rising number of publications on their effects on workers (Bock et al., 2022). A systematic review and meta-analysis by Bär et al. (2021) summarized the results of recent studies and reported effects of exoskeletons on physical strain and stress (Bär et al., 2021). Similarly, a systematic review by Kermavnar et al. (2021) reported the effects of back exoskeletons on body loading and user experience (Kermavnar et al., 2021).

The existing evidence suggests that exoskeletons can potentially reduce WMSD risk. Further, the studies revealed that exoskeletons tend to show more of their potential in static activities, while in dynamic tasks, they can hinder regular job performance (Baldassarre et al., 2022). Thus, more research is required to understand how exoskeletons can be adapted to different tasks and work environments. The majority of these studies focused on the evaluation of biomechanical (e.g., muscle activity or joint angles) and subjective measures (e.g., perceived exertion or discomfort). However, Del Ferraro et al. (2020) suggest that it is equally important to investigate physiological effects of the exoskeleton on the user. One of their arguments in favor is that the additional weight of the exoskeleton might increase energy expenditure during the performed activities. Currently, the body of knowledge on physiological effects is limited, compared to biomechanical effects (Del Ferraro et al., 2020). A recent review by Del Ferraro et al. (2020) summarized nine studies and revealed significant reductions on metabolic and cardio-respiratory measures when wearing upper-body exoskeletons. Due to the limited number of studies, the results must be considered preliminary and thus require further investigations.

Besides biomechanical and physiological benefits, exoskeletons are touted to have a positive impact on productivity (Howard et al., 2019). However, this cannot be confirmed due to lack of data (Kermavnar et al., 2021). Additionally, Kaupe et al. (2021) identified a lack of studies in the logistics sector. Since this sector is among the occupational sectors with the highest prevalence of backache, exoskeleton use might be especially beneficial.

Thus, this study aimed to evaluate a commercial passive low-back exoskeleton in a simulated logistics environment. Objective measures include heart rate, trunk inclination, trunk acceleration, and throughput (as a measure of productivity). Subjective measures comprise perceived exertion, usability, and acceptance.

## 2. Methods

### 2.1. Participants

Thirty participants (22 men and 8 women) were recruited for this laboratory study using convenience sampling. Mean (SD) age, height, and body weight were 29.0 (8.3) years, 180.2 (9.1) cm, and 74.8 (12.6) kg, respectively. Eighteen participants reported to have experienced pain or discomfort in either the hip, lower back, or upper back within the last year prior to the study. Further, eight participants reported to have experienced pain or discomfort in at least one of these body regions within 7 days prior to the study. Participants were either university students (*n* = 26) or white-collar workers (*n* = 4). None of them had any relevant experience with exoskeletons. Informed consent was obtained from all participants prior to the study.

### 2.2. Exoskeleton

**Figure 1.**
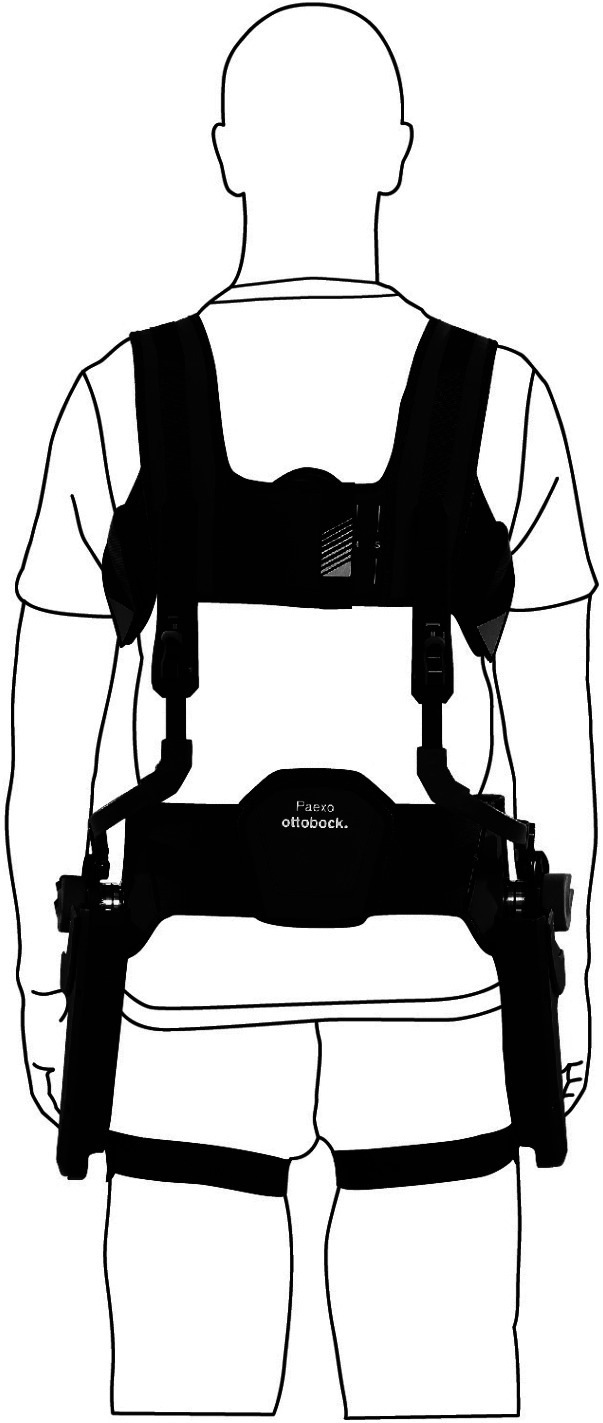
Paexo Back from Ottobock (© Ottobock).

The study evaluated the effects of the Paexo Back from Ottobock (Ottobock SE & Co. KGaA, 2021), which is depicted in Figure 1. The Paexo Back is a commercial passive exoskeleton, providing physical support to the lower back. Like a backpack, the load is taken off at the shoulder and redirected to the thighs with the help of the exoskeleton’s support structure. The energy store absorbs force when bending and releases it again when lifting. The support force can be adjusted by a control knob. The exoskeleton is aimed at logistics companies and warehouses as a measure to support their employees in manual material handling.

### 2.3. Workplaces

**Figure 2.**
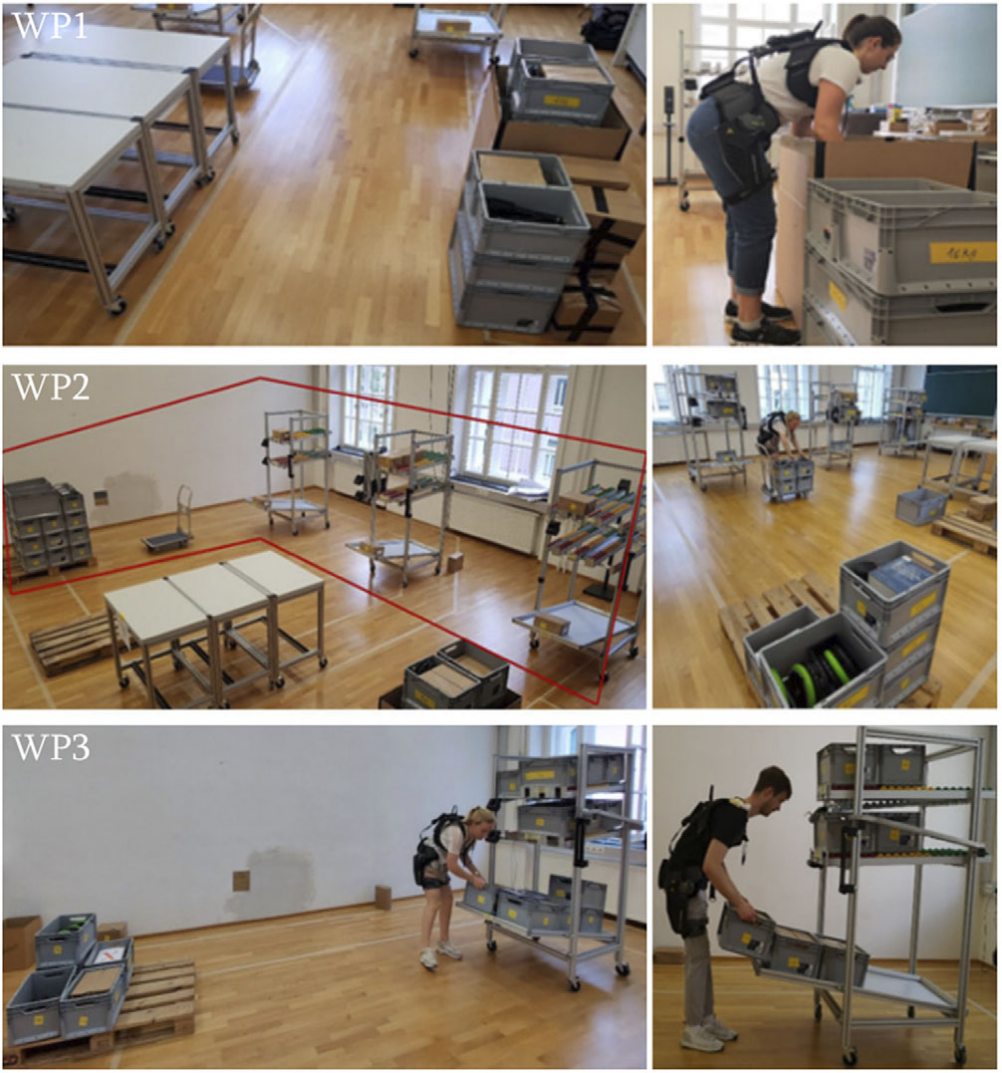
Setup of the workplaces in the laboratory.

In a laboratory environment, three workplaces that aim to imitate typical logistics tasks were established. Tasks included lifting, holding, carrying, bending, pushing, pulling, and walking. The weights of the handled goods were randomly determined between 3 and 20 kg, since this is a typical range of weights occurring in logistics (Hensel and Keil, 2019; Kaupe et al., 2021). Mean weight was 11.3 kg for men and 9.1 kg for women, respectively. An overview of the workplaces is depicted in Figure 2.

Workplace 1 (WP1) simulated an unloading and loading station of goods. It required participants to pick goods from a deep box (depth: 0.8 m) and place them onto a table (height: 0.9 m) that was positioned 1.5 m behind them. Subsequently, participants returned all goods into the box.

Workplace 2 (WP2) imitated order picking and storing with a man-to-goods principle. Three racks were aligned in a row, where each rack had shelf spaces for three different weights. Heavier goods were stored in the lower shelves, whereas lighter goods were stored in the higher shelves. Racks, shelf spaces, and goods were labeled systematically (e.g., A1 for the highest shelf of the first rack and C3 for the lowest shelf of the last rack). First, participants picked up goods from the incoming goods area and placed them on a trolley (six goods at a time). Second, participants pushed and pulled the trolley to the designated rack and shelf space to store the goods. Following storing of the goods, participants were provided with an order list. According to this list, they placed goods from the shelf spaces onto the trolley and unloaded them in the outgoing goods area (identical to the incoming goods area). The order list consisted of four orders, which were completed one after another.

Workplace 3 (WP3) simulated a simple storing task. Participants were required to pick up goods from the incoming goods area, walk 4 m to the first rack (A1–A3), and store the goods in the rack. Following storing all goods, the task was reversed.

Ergonomic risk at the workplaces was assessed using the Key Indicator Method for lifting, holding, and carrying and Key Indicator Method for pushing and pulling movements (Steinberg, 2012). The resulting risk score corresponds to the risk for overload, where an increasing score implies higher stress on the musculoskeletal system. WP 1 and WP 3 were considered increased load situation (i.e., physical overload is possible for less resilient persons) and WP 2 is considered a low load situation (i.e., physical overload unlikely to appear).

### 2.4. Study design

Cross-over research design was utilized to evaluate the effects of the exoskeleton. The study consisted of a control (NE) and an intervention (E) condition. Each condition lasted approximately 25 min. Between the conditions, a 20-min resting period was implemented where the participants were asked to remain seated. The starting condition was assigned randomly for each participant, and the allocation sequence was concealed until participants were enrolled and assigned to starting condition.

Prior to the starting condition, demographic information and musculoskeletal complaints were obtained from each participant by a baseline questionnaire. Further, the resting frequency was measured and the maximum heart rate estimated. Prior to the intervention condition, participants were introduced to the exoskeleton. Then, the exoskeleton was adjusted for each individual participant by a trained individual and participants were given approximately 10 min to familiarize and readjust it if deemed necessary. Within this period, participants chose their preferred support level for the upcoming session.

Concluding each condition, participants were asked to provide information on perceived exertion. Following the intervention condition, they were also asked to provide information on usability and acceptance of the exoskeleton.

During both conditions, participants worked on all three workplaces in succession. First, they worked at WP1 for 4 min. Second, they worked at WP2 for 10 min. Last, they worked at WP3 for 6 min. Participants were verbally encouraged to work in a speed they believe they could sustain throughout an entire workday.

### 2.5. Data collection

Demographic information and musculoskeletal complaints were obtained by utilizing parts of the Nordic questionnaire (Kuorinka et al., 1987). Additionally, participants were asked to provide information on their physical activity (i.e., hours of physical activity performed in daily life).

Physiological data (i.e., heart rate) and biomechanical data (i.e., trunk inclination and three-dimensional acceleration) were captured using the Zephyr Bioharness 3.0. The Zephyr Bioharness is a wireless chest-based physiological monitoring telemetry device (Zephyr Technology, 2012), which has demonstrated reliability and validity in previous field testings (Johnstone et al., 2012; Nazari et al., 2018). The sensor pads were moistened, and the chest strap was placed around participants’ chest prior to the study. To measure the resting heart rate, participants were asked to remain seated in a relaxed upright position for 5 min. Resting heart rate was defined as the average heart rate during the last minute. Maximum heart rate was estimated ( 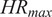 ) using the following formula proposed by Malchaire et al. (2017): 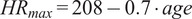 (Malchaire et al., 2017).

Heart rate, trunk inclination, and acceleration were measured continuously throughout each trial. Sample rate was 1 Hz for heart rate and trunk inclination, and 100 Hz for acceleration. The Zephyr Bioharness internally averages the three axial acceleration magnitudes over the previous second and thus reporting frequency was 1 Hz.

Similar to a previous study by Kim et al. (2021b), perceived exertion was administered using two adopted statements from the psychological climate and effort measures questionnaire (Q1: “When I work, I really exert myself to the fullest” and Q2: “I feel exhausted at the end of this trial”) (Brown and Leigh, 1996). Participants were asked to respond to each statement on a 100-mm Visual Analog Scale (VAS) from 0 (strongly disagree) to 100 (strongly agree). The average score of both questions (i.e., combination of perceived intensity and exhaustion) is considered the perceived exertion.

The questions on usability and acceptance are displayed in Table 1. Usability was obtained on a 7-point UMUX-Lite scale (Lewis et al., 2013), which is a shorter alternative questionnaire to obtain the same information as the System Usability Scale (SUS) (Brooke, 1995). Scores were adjusted using linear regression (Lewis et al., 2015) to match the SUS. Acceptance was assessed on a 7-point scale with parts of the Technology Acceptance Model 2 (Venkatesh and Davis, 2000). Also, acceptance was further assessed by two questions regarding perceived wearing comfort and movement restrictions from a custom questionnaire by Kim et al. (2021b) (Q1: “What was your perception of overall wearing comfort?” and Q2: “Did you feel that your range of motion was limited while wearing the exoskeleton?”). Both questions were administered using a 100-mm VAS from 0 (no comfort/restrictions) to 100 (extremely comfortable/restricting). The questionnaires were self-administered and completed by each participant. All questionnaires were originally in German. For this article, all questions were translated to English, without affecting the results.

**Table 1.** Usability and perceived usefulness scores for all participants (*n* = 30). SD indicates the standard deviation, and IQR indicates the interquartile range. All items were measured on a 7-point scale, where 1 = strongly disagree, 2 = moderately disagree, 3 = somewhat disagree, 4 = neutral (neither disagree nor agree), 5 = somewhat agree, 6 = moderately agree, and 7 = strongly agree. Usability scores were adjusted using linear regression to match the System Usability Scale (SUS)

Item text	Points on a 7-point Likert Scale			
	Mean	SD	Median	IQR
Perceived usability (UMUX-Lite)				
The exoskeleton’s capabilities meet my requirements.	4.5	1.3	5.0	4.0–5.0
The exoskeleton is easy to use.	5.6	1.2	6.0	5.0–6.0
**Overall usability (SUS score)**	**66.4**	**11.0**	**66.2**	**60.8–71.7**
Perceived usefulness (PU)				
Using the system improves my performance in my job.	4.4	1.2	4.5	4.0–5.0
Using the system in my job increases my productivity.	4.3	1.2	4.0	4.0–5.0
Using the system enhances my effectiveness in my job.	4.1	1.4	4.0	3.0–5.0
I find the system to be useful in my job.	4.7	1.7	5.0	3.3–6.0
**Overall perceived usefulness**	**4.4**	**1.2**	**4.5**	**3.5–5.0**

Finally, throughput was measured by manually counting the number of picks per workplace.

### 2.6. Data analysis

Python 3.8 was used for offline data processing. Exoskeleton effects were explored across the individual tasks; thus, data for each workplace were extracted from the dataset of each participant. Raw heart rate data were normalized to percentages of participants’ heart rate reserve (% HRR). Thus, normalized heart rate ranges from 0% HRR (heart rate equal to resting heart rate) to 100% HRR (heart rate equal to maximum heart rate).

Three-dimensional acceleration data were internally averaged by the sensor and did not require further processing. Similarly, trunk inclination (i.e., physical positioning of the torso in relation to the *X*- and *Y*-axes) was derived internally by the Zephyr Bioharness based on acceleration data. Further, trunk inclination was divided into three well-established ranges (International Organization for Standardization, 2000; European Committee for Standardization, 2008): (1) <20°, (2) 20–60°, and (3) >60°. Time spent in each of these ranges was calculated.

IBM SPSS Statistics 28.0 was used for the statistical analysis of the results. Significance level was set to 95%. Data were checked for normal distribution using the Shapiro–Wilk test. Paired-samples *t*-test was used to compare means between the conditions. Independent-samples *t*-test was used to investigate the impact of human factors. In particular, we investigated the effect of gender and musculoskeletal complaints on trunk kinematics and user experience. Further, we explored potential differences in age, height, weight, and physical activity between participants with a heart rate decrease when wearing the exoskeleton, compared to participants with a heart rate increase. Lastly, we examined whether changes in throughput can be linked to physical activity of the participants.

## 3. Results

### 3.1. Heart rate

**Figure 3.**
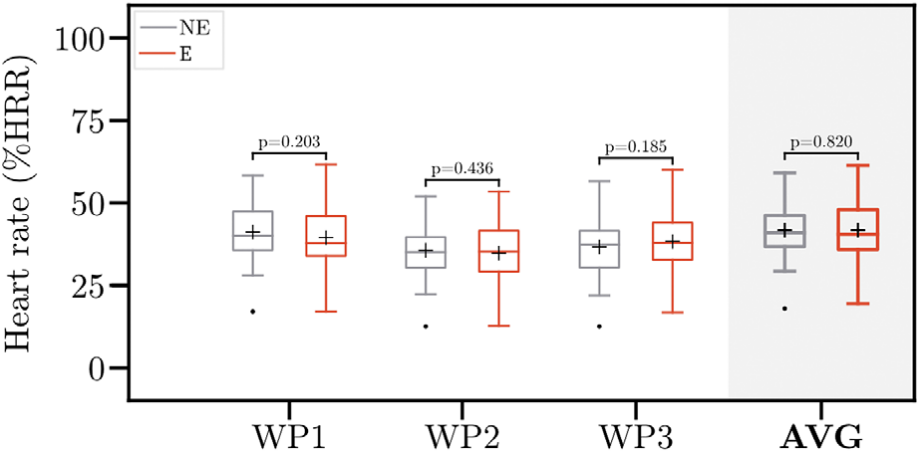
Normalized heart rate without (NE) and with (E) the exoskeleton. Boxplots comprise data from 30 participants. Whiskers are set to 1.5  IQR (interquartile range). Points indicate the outliers. Crosses indicate the mean normalized heart rate. HRR, heart rate reserve; WP1–3, Workplace 1–3; AVG, averaged normalized heart rate across all workplaces.

Figure 3 depicts the normalized heart rate values across the workplaces. The averaged heart rate ranges from 14.0%HRR to 55.2%HRR without wearing the exoskeleton and from 15.5%HRR to 57.5%HRR when wearing the exoskeleton, respectively. The mean (SD) averaged heart rate across all workplaces without wearing the exoskeleton is 37.8 (8.6)%HRR, compared to 37.5 (9.8)%HRR when wearing the exoskeleton. This corresponds to a non-significant reduction in averaged heart rate by 0.8% when wearing the exoskeleton.

On the workplace level, the mean normalized heart rate decreased by 3.6% at WP1 and 1.7% at WP2, respectively. In contrast, at WP3, participants experienced an increase in mean normalized heart rate by 5.9%. None of the differences yields statistical significance.

Overall, normalized heart rate values varied widely between participants. Also, the effects of the exoskeleton on the heart rate differed highly between participants. Sixteen (12 males and 4 females) participants experienced a decrease in normalized heart rate, whereas 14 (10 males and 4 females) experienced an increase. Independent-samples *t*-test did not reveal any significant differences in weight (*p* = .260), height (*p* = .792), and physical activity (*p* = .255) between these two groups. Participants experiencing a decrease in heart rate were slightly older (31.6 vs. 26.1 years). However, the difference is not statistically significant (*p* = .065).

### 3.2. Trunk kinematics

Due to a systematic measurement error, trunk kinematics had to be discarded for nine participants. Thus, final results are limited to 21 participants (14 men and 7 women).

**Figure 4.**
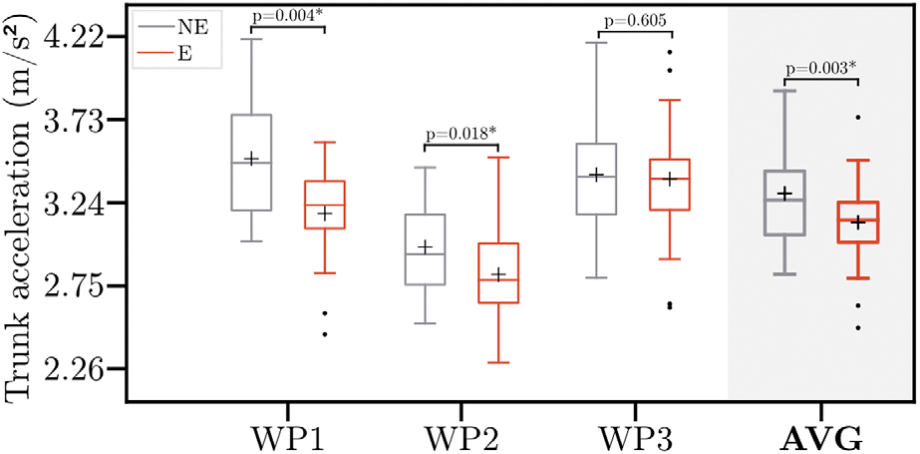
Trunk acceleration magnitudes without (NE) and with (E) the exoskeleton. Boxplots comprise data from 21 participants. Whiskers are set to 1.5  IQR (interquartile range). Points indicate the outliers. Crosses indicate the mean trunk acceleration. Asterisk indicates the significant difference (*p* < .05). WP1–3, Workplace 1–3; AVG, averaged trunk acceleration across all workplaces.

Figure 4 depicts the trunk acceleration across the workplaces. On average, the trunk acceleration reduced significantly by 5.0% (*p* = .003) when wearing the exoskeleton across nearly all participants (85.7%). No differences in exoskeleton effects on acceleration were revealed between gender (*p* = .690) and health status (*p* = .364). Regarding workplaces, no significant differences in trunk acceleration were observed at WP3. Contrary, at WP1 and WP2, the mean trunk acceleration reduced significantly by 8.0% (*p* = .004) and 5.2% (*p* = .018), respectively.

**Figure 5.**
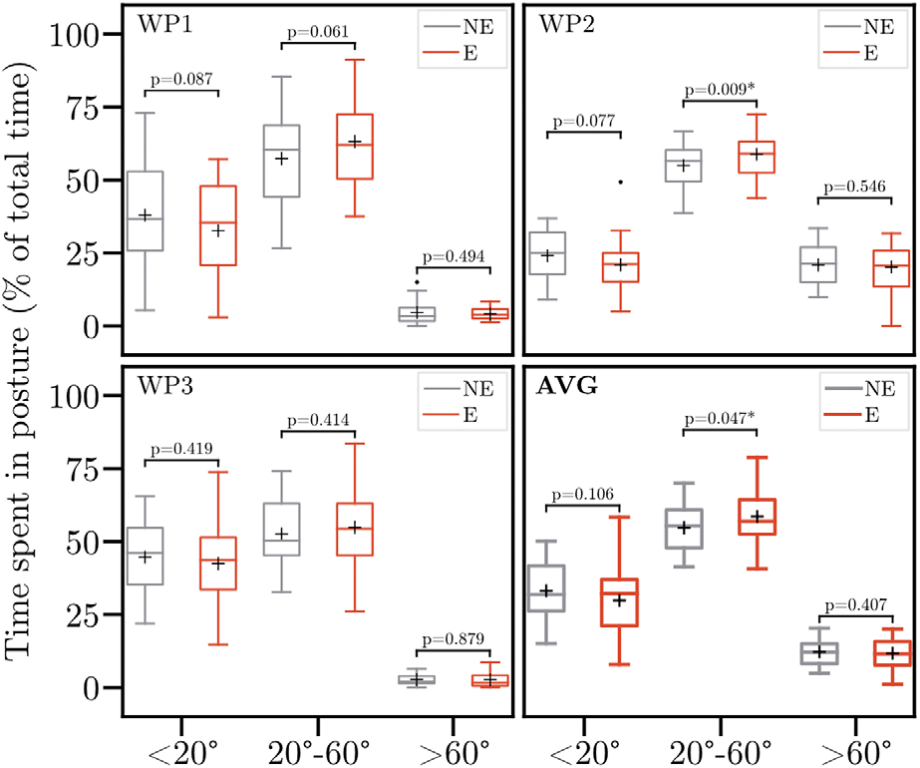
Trunk inclination (time spent in posture) without (NE) and with (E) the exoskeleton. Boxplots comprise data of 21 participants. Whiskers are set to 1.5  IQR (interquartile range). Points indicate the outliers. Crosses indicate the mean trunk inclination. Asterisk indicates the significant difference (*p* < .05). WP1–3, Workplace 1–3; AVG, averaged trunk inclination across all workplaces.

Regarding trunk inclination, times spent within the pre-defined ranges are shown in Figure 5. At WP2, wearing the exoskeleton resulted in a significant increase for time spent between 20° and 60° (*p* = .009). However, no significant differences were observed for WP1 and WP3. Across all workplaces, the trunk inclination predominately ranged between 20° and 60° (on average, 56.9% of total time). Further, time spent below 20° and above 60° comprised on average 33.2 and 9.9% of the total time, respectively. Wearing the exoskeleton caused a significant 6.9% increase in the time spent between 20° and 60°. The time spent below 20° and above 60° decreased by 10.0 and 4.2%, respectively. However, none of these decreases is statistically significant.

### 3.3. Throughput

**Figure 6.**
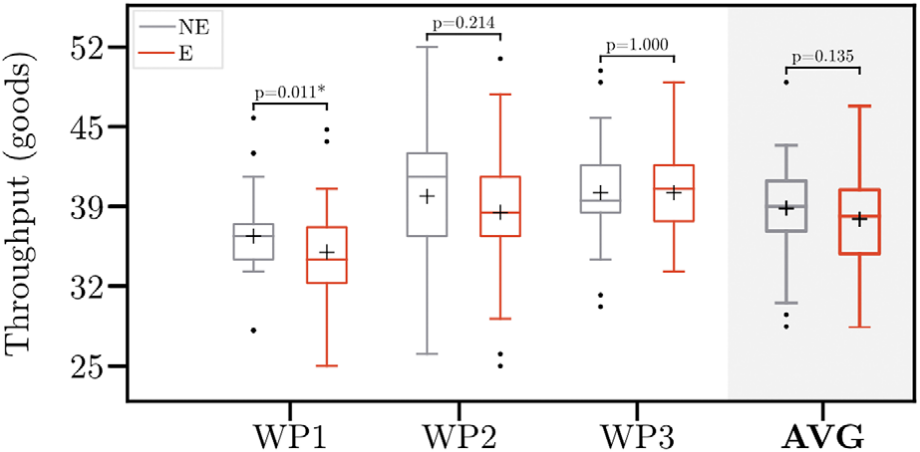
Throughput without (NE) and with (E) the exoskeleton. Boxplots comprise data of 30 participants. Whiskers are set to 1.5  IQR (interquartile range). Points indicate the outliers. Crosses indicate the mean throughput. Asterisk indicates the significant difference (*p* < .05). WP1–3, Workplace 1–3; AVG, averaged throughput across all workplaces.

Figure 6 depicts the throughput across all workplaces. Throughput varied widely between the participants. While it increased for 12 (9 males and 3 females) participants, throughput decreased for 18 participants (13 males and 5 females). Overall, wearing the exoskeleton reduced mean throughput across all workplaces by 1.9%. This corresponds to one pick within 20 min, which is statistically not significant. However, at WP1, throughput reduced significantly (*p* = .011) by 3.6%, which corresponds to two picks within 4 min. Independent-samples *t*-test revealed that participants experiencing an increase in throughput engage in more physical activity (7.9 vs. 4.5 hr per week, *p* = .041).

### 3.4. User experience

**Figure 7.**
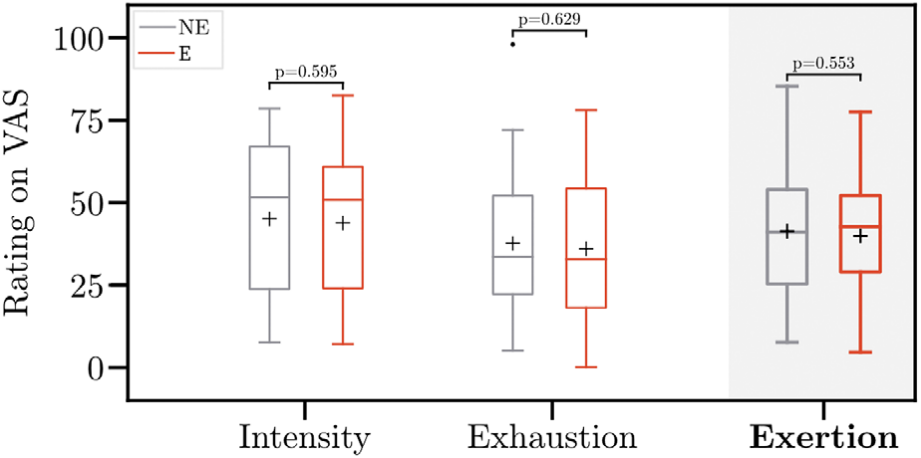
Perceived exertion without (NE) and with (E) the exoskeleton. Ratings for perceived exertion represent the average ratings for perceived intensity and exhaustion. Boxplots comprise data of 30 participants. Whiskers are set to 1.5  IQR (interquartile range). Points indicate the outliers. Crosses indicate the mean values. Asterisk indicates the significant difference (*p* < .05). VAS, Visual Analog Scale.

Figure 7 depicts the VAS ratings on perceived exertion. Work intensity (Q1: “When I work, I really exert myself to the fullest”) was rated 45.0 without versus 43.8 with wearing the exoskeleton. Further, exhaustion (Q2: “I feel exhausted at the end of this trial”) was rated 37.6 and 35.9 without and with wearing the exoskeleton, respectively. On average, the ratings for perceived exertion were 41.3 without wearing the exoskeleton, compared with 39.8 with wearing the exoskeleton. No significant differences between the conditions were observed. Also, the independent-samples *t*-test revealed no differences in perceived exertion between gender (*p* = .973) and people with and without musculoskeletal complaints (*p* = .479).

Table 1 presents the results for perceived usability and usefulness following the intervention phase. Items were measured on a 7-point scale, where 1 = strongly disagree and 7 = strongly agree. On average, usability was rated 66.4 out of 100 points. Women rated usability slightly higher than men (70.3 vs. 65.0, *p* = .249). Perceived usefulness was rated 4.4 on the 7-point scale. Women and people with musculoskeletal complaints rated perceived usefulness higher compared with men (4.9 vs. 4.2, *p* = .159) and people without musculoskeletal complaints (4.8 vs. 4.2, *p* = .188), respectively. However, none of these differences are statistically significant.

**Figure 8.**
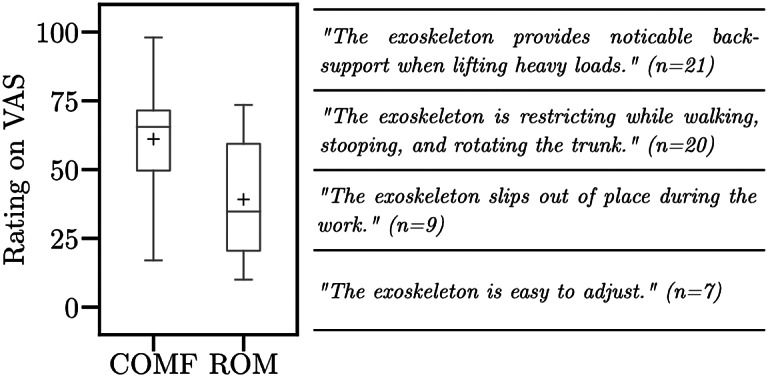
Perceived wearing comfort (COMF) and movement restrictions (ROM) when wearing the exoskeleton. Boxplots comprise data of 30 participants. Whiskers are set to 1.5  IQR (interquartile range). Points indicate the outliers. Crosses indicate the mean rating on VAS. The statements next to the boxplots represent the most common remarks by participants on the exoskeleton. VAS, Visual Analog Scale.

Additionally, Figure 8 depicts the ratings for perceived wearing comfort and movement restrictions. Wearing comfort was rated 63.3 out of 100 points. Further, the movement restrictions due to the exoskeleton were rated 41.3 of 100 points. Next to the boxplots, the two most frequent positive and negative remarks by the participants are displayed. Contrary to the VAS rating, 20 participants remarked that the exoskeleton is restricting while walking, stooping, and rotating the trunk. Regarding comfort, the most frequently raised concern regarded the fact that the exoskeleton slips out of place during work. Also, seven participants raised concerns that wearing the exoskeleton increases sweating, especially at the contact points. Still, participants felt that the exoskeleton provides noticeable back support (*n* = 21) and is easy to adjust (*n* = 9).

Following the trial, 23 participants indicated that they intend to use the exoskeleton in the future (assuming that they work in logistics). Also, these participants rated perceived usability and usefulness significantly higher (*p* = .001 and *p* = .002, respectively) than participants without intention to use.

## 4. Discussion

The laboratory study yielded mostly non-significant group differences between wearing and not wearing the exoskeleton. However, results varied widely between participants and tasks. This confirms that exoskeleton effects depend highly on the user and the task (Omoniyi et al., 2020; Schmalz et al., 2021).

### 4.1. Heart rate

The normalized heart rate of each participant was assessed across all workplaces to estimate energy expenditure. Recommendations by the World Health Organization suggest that the average %HRR during an 8-hr working day should not exceed 30% (Wu and Wang, 2002; Astrand et al., 2003). Workloads above this limit pose a substantially increased risk for cardiovascular disorders (Dinh-Dang et al., 2023). In our study, this limit was exceeded by almost all participants (86.7%). These results match with the estimated KIM risk scores per workplace, suggesting that workplace redesign and other prevention may be helpful, especially for less resilient persons.

Consistent with previous studies, average heart rate did not change significantly. Schmalz et al. (2021) found no significant difference in heart rate when evaluating a passive back-support exoskeleton for manual materials handling. Similarly, Godwin et al. (2009) did not observe changes in heart rate when testing the efficacy of an ergonomic lifting aid (Godwin et al., 2009).

While the average difference in heart rate seems negligible, the results varied wildly between the participants. One participant experienced an increase in heart rate by almost 30% when wearing the exoskeleton, whereas for another person, heart rate decreased by more than 36%. Similar trends were observed by Schalk et al. (2022), who attributed this to the body composition of the participants. Our data do not suggest a relationship between body composition and effects on heart rate. Future research should investigate the relationship between exoskeleton effects and age.

Analyzing heart rate at the individual workplaces, we found that the results varied widely. The results suggest that a heart rate increases at WP3, which might be attributed to the workplace characteristics. WP3 simulated a storing task that required more walking, compared to other workplaces. Baltrusch et al. (2019a) showed that walking with an exoskeleton increases metabolic costs up to 17%. One possible explanation for this increase might be the weight of the exoskeleton, which weighs approximately 4 kg. Also, the participants frequently remarked that it gets very hot under the exoskeleton. The increasing heat and perspiration might have attributed to an elevation in heart rate. This suggests that the exoskeleton only provides support during lifting and static bending. Further, it potentially interfered with the participants during carrying (Poliero et al., 2020), pulling (Lazzaroni et al., 2021), and walking (Park et al., 2022). Future studies might further explore exoskeleton interference with task execution.

In general, we agree with Schalk et al. (2022) that heart rate likely seems unsuitable to assess short-term effects of exoskeletons. However, Schalk et al. (2022) found significant differences in the slope of the heart rate over a 30-min period. This suggests that investigations of physiological effects require long-term studies. Therefore, future research in this direction might yield novel findings.

### 4.2. Trunk kinematics

Across all workplaces, time spent in an upright (trunk inclination below 20°) and strongly bent forward position (trunk inclination above 60°) is reduced with the exoskeleton. Consequently, time spent bent forward between 20° and 60° increased. On average, time spent between 20° and 60° increased from 54.7 to 58.5% of total time, yielding statistical significance (*p* < .05). However, the effects of this increase remain uncertain since the exoskeleton is designed to provide trunk extension support and theoretically reduce loading in the lower back.

Simultaneously to the inclination, mean trunk acceleration dropped significantly, especially for WP1 (i.e., bending to the floor of a deep box). This reduction in acceleration might be possibly attributed to individual preferences to work speed, since participants were instructed to work at their own preferred pace. The subjective feedback of the participants provides another possible explanation for these changes. One third of the participants reported being restricted in movement by the exoskeleton. This restriction potentially reduced acceleration. Consequently, more time is spent in a bent-forward posture. However, these changes have no impact on the KIM risk scores.

Our results do not differ substantially from previous studies. Dewi and Komatsuzaki (2018) observed reduced trunk acceleration values during commercial farming tasks when wearing a low-back exoskeleton. Regarding trunk inclination, Motmans et al. (2018) reported a 3% increase in back flexion above 30° when wearing an exoskeleton during order picking in logistics. Kim et al. (2020) found small and inconsistent changes in trunk inclination angle during manual assembly tasks. Similarly, Baltrusch et al. (2019b) and Graham et al. (2009) observed no changes in trunk inclination during symmetric lifting or tasks in a car assembly, respectively. Contrary to our results, none of the aforementioned studies yielded statistical significance. However, it remains arguable whether these statistical differences are of any practical relevance. Especially, since our results are limited to the data collected from one acceleration sensor positioned at the chest strap. Further, ergonomic assessment tools often lack the gradation to reflect such minor changes. Therefore, future research should capture whole-body kinematics to enhance information on spinal curvature and rotational movements.

### 4.3. Throughput

On average, throughput (i.e., number of picks) did not change significantly when wearing the exoskeleton. However, effects varied between workplaces. At WP1, we observed a decrease by two picks within the 4-min period. This might be related to the reduction in trunk acceleration for this workplace. Assuming constant working speed (which we consider valid because participants were encouraged to work at a sustainable working speed) throughout an 8-hr shift at this workplace, this results in a decrease of 120 picks. Schwerha et al. (2022) also reported that workers slowed down due to an exoskeleton and could no longer meet their productivity goals. Contrary to these results, Miura et al. (2018) found that workers’ performance increased significantly when wearing an active lumbar support exoskeleton. In addition, Dewi and Komatsuzaki (2018) found that productivity was not affected by the exoskeleton but significantly differed between gender. While we did not observe differences between gender, our results revealed a significant impact of physical activity on productivity effects of exoskeletons. At WP2, throughput varied widely between participants. Likely, this is caused by the different commissioning strategies chosen by participants. While some participants memorized the picking list, others read it prior to each pick.

To date, no standardized procedure to assess the impact of exoskeletons on productivity exists. However, our results suggest that it is insufficient to evaluate isolated tasks. In 2010, de Looze et al. (2010) revealed that productivity gains at individual workplaces often cannot be exploited, since subsequent processes are not designed for the increasing volumes. Therefore, we suggest that the evaluation of exoskeleton effects on productivity should always follow a holistic approach.

### 4.4. User experience

Ratings on perceived exertion suggest a moderate workload, which confirms the objective output measures (i.e., heart rate and KIM risk score). Participants frequently reported that they felt a stress reduction in the low back due to the exoskeleton. This is indicated by the ratings on perceived usefulness. However, perceived exertion did not differ significantly between wearing and not wearing the exoskeleton. These results agree with findings from previous studies (Cardoso et al., 2020; Kim et al., 2021a). Possibly, the workload was not sufficiently exhausting to trigger significant differences across all participants.

Overall, usability of the exoskeleton was rated 66.4. Bangor et al. (2008) suggest that systems with a SUS score below 68 are considered poor and with a score above 85 excellent. Thus, usability of the exoskeleton was considered poor among participants. Frequent complaints were raised regarding donning and doffing. According to Hoffmann et al. (2019), time and effort required for donning and doffing impacts perceived usability significantly. Likely, the poor usability score is also attributed to the rather short training period. A study by Steinhilber et al. (2020) suggests that extensive instructions and regular checkups might increase usability and acceptance. Thus, our observed results might not indicate actual usability and acceptance. To the best of our knowledge, this study was the first to investigate the usability of the Paexo Back. Thus, comparison to previous studies is impossible.

Perceived wearing comfort and movement restrictions appear to be key determinants for exoskeleton intention to use (Kim et al., 2021a). Thus, both measures were assessed in this study. However, we found no significant difference between participants with and without intention to use the exoskeleton in the future. Rather, the study revealed large differences between the participants, especially concerning comfort. Many participants felt uncomfortable because the exoskeleton slipped out of place during work. Also, many participants raised concerns regarding the heat below the exoskeleton. Like usability, it seems plausible that these ratings remain valid to a limited extent and participants require more time to establish true perception. Surprisingly, remarks by the participants contradict with their ratings regarding perceived movement restrictions. While two thirds of the participants remarked that the exoskeleton is restricting, VAS ratings suggest the opposite. Potentially, the restriction was too small to have an impact on the VAS ratings.

Further, we investigated the relationship between human factors and user experience. Our results revealed a trend that women and people with musculoskeletal complaints perceived the exoskeleton more useful. Similarly, a study by Baltrusch et al. (2020) showed that exoskeleton use slightly increases self-efficacy in workers with low-back pain. Future research might consider participants with musculoskeletal complaints to further explore the differences between exoskeleton effects on them and healthy participants.

Finally, we found that participants with intention to use the exoskeleton in the future experienced a significantly larger reduction in perceived exertion than participants without intention to use (*p* = .015). Future studies might further explore the relationship between intention to use and perceived exertion.

### 4.5. Strengths and limitations

Due to current COVID-19 regulations, the study could not be conducted in the field as planned. However, according to Schmalz et al. (2021), experimental setups that closely replicate real workplaces are an appropriate approach for evaluating exoskeletons. Yet we must acknowledge certain limitations of this study. First, this study evaluated the effects of only one exoskeleton and results are likely not generalizable to other systems. Second, exoskeleton usage was limited to approximately 20 min, rendering conclusions on long-term effects impossible. Third, initial training with the exoskeleton was rather short. Fourth, results on trunk kinematics were limited to one acceleration sensor. Thus, no information on spinal curvature and rotational movements was available. Further, musculoskeletal pain experienced by the participants was not controlled in the study. Thus, acceleration data were possibly altered. Lastly, participants were predominantly healthy young men with no prior experience in logistics. Consequently, the results might not be the representative of professional logistics workers.

However, there are also noteworthy strengths of this laboratory study due to which we believe that it contributes to a better understanding of the effects of industrial exoskeletons in the logistics sector. To the best of our knowledge, this study is among the first to evaluate the effects of an industrial exoskeleton on heart rate and productivity during simulated logistics tasks. Using an objective measure of energy expenditure (%HRR) instead of subjective measures is the major strength of this study. Also, rather than evaluating an isolated task, we aimed to imitate three realistic logistics tasks and added secondary activities, like walking and reading a picking list.

## 5. Conclusion

This study evaluated a commercial passive low-back exoskeleton during simulated logistics tasks. The evaluation included user experience and effects of the exoskeleton on heart rate, trunk kinematics, and throughput. We found mostly non-significant differences between wearing and not wearing the exoskeleton. However, results varied widely between participants and workplaces. The findings suggest that exoskeleton effects depend highly on the ideal fit between the task and the exoskeleton design.

While statistically significant, the effect of postural changes on ergonomic risk is unknown, especially when factoring in trunk support from the exoskeleton. Throughput significantly decreased for one workplace when wearing the exoskeleton, while remaining statistically similar for the other two workplaces. Concerning user experience, perceived exertion did not change significantly. Women and participants with musculoskeletal complaints rated perceived usefulness slightly higher than their counterparts. While most participants felt assisted by the exoskeletons, they raised concerns regarding movement restrictions and donning and doffing. It remains unclear whether these concerns were raised due to lack of training or the exoskeleton design. Concluding, our results suggest that a holistic evaluation and implementation approach for industrial exoskeletons is necessary. Also, prior to exoskeleton implementation, adaptation of workplaces might be required to obtain the ideal fit between the tasks performed in the workplace and exoskeleton characteristics. On the other hand, exoskeletons that assist different tasks may be more suitable for an unstructured working environment characterized by non-predictable and non-repetitive tasks.

We believe that this study enhances knowledge concerning industrial exoskeleton. When evaluating and implementing exoskeletons, practitioners should apply a holistic approach. Exoskeleton design should be adapted to reduce contact points to participants and simplify donning and doffing. Future research should focus on in-field evaluation of exoskeletons at dynamic workplaces with actual workers. These studies should also cover active exoskeletons, since they can assist the user also during tasks like holding, pushing, and walking. Further, long-term effects on physiological parameters might be addressed by future research. Lastly, research on the time required to acquire a stable perception about the exoskeleton seems necessary.

## Data Availability

The data that support the findings of this study are available from the corresponding author (M.W.) upon reasonable request.
